# *Ganoderma applanatum* mushroom provides new insights into the management of diabetes mellitus, hyperlipidemia, and hepatic degeneration: A comprehensive analysis

**DOI:** 10.1002/fsn3.2407

**Published:** 2021-06-24

**Authors:** Mohammad Shahadat Hossain, Anik Barua, Mohammad Akramul Hoque Tanim, Mohammad Sharif Hasan, Mohammad Jahedul Islam, Md. Rabiul Hossain, Nazim Uddin Emon, S M Moazzem Hossen

**Affiliations:** ^1^ Department of Pharmacy Faculty of Biological Science University of Chittagong Chattogram Bangladesh; ^2^ Department of Biochemistry and Biotechnology University of Science and Technology Chittagong Chattogram Bangladesh; ^3^ Department of Pharmacy University of Science and Technology Chittagong Chattogram Bangladesh; ^4^ Department of Pharmacy Faculty of Science and Engineering International Islamic University Chittagong Chattogram Bangladesh

**Keywords:** diabetes mellitus, *Ganoderma applanatum*, hypolipidemic, hepatoprotective, mushroom

## Abstract

This study was undertaken to evaluate the antidiabetic, hypolipidemic, and hepatoprotective effects of methanol and aqueous extracts of *Ganoderma applanatum* (MEGA, AEGA) in alloxan‐induced diabetic rats. The antidiabetic study was implemented by the induction of alloxan to the rats. The analysis of the hypolipidemic and liver‐protective effects of fungus extracts was studied by estimating the lipid profile and the liver marker enzymes. Besides, in silico screening of the compounds of *Ganoderma applanatum* has been incorporated thus to check the binding affinity of compounds and enzymes affinity. The Discovery Studio 2020, UCSF Chimera, and PyRx AutoDock Vina have been used to implement the docking analysis. Nine days of oral feeding of MEGA and AEGA of *Ganoderma applanatum* resulted in a significant (*p* < .001) reduction in blood glucose, lipid profile, and liver marker enzymes. Besides, Myrocin C scored the highest score in the docking study. The biological and computational approaches suggested the MEGA and AEGA could be a potential source for antidiabetic, hypolipidemic, and hepatoprotective effects.

## Graphical abstract

This study aggregates, consolidates, and validates the use of mushroom for the treatment of diabetes, hyperlipidemia, and hepatic degenerations, Besides, the bioactive compounds have been accelerated to investigate the binding reactions with diabetes, hyperlipidemia, and hepatic receptors.

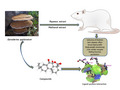

## 1 INTRODUCTION

Diabetes mellitus is a metabolic disorder characterized by a relative or total insulin deficit and hyperglycemia (Karunasagara et al., 2020). Diabetic patients suffer from numerous disorders, including vascular illness, like atherosclerosis, neuropathic pain, and diabetes neuropathy (Papatheodorou et al., 2018). According to the report of 2019, 463 million individuals between the ages of 20 and 79 are estimated to have diabetes globally (Saeedi et al., 2019). By 2045, the number of affected people with diabetes is expected to increase to 700 million worldwide. Bangladesh has been in the top ten positions in the world for the number of adults (20–79 years) with diabetes (8.4 million) in 2019. The prevalence of diabetes will be increased to 15.0 million by 2045. Bangladesh has also been ranked nine in 2019 among the countries for 4.7 million adults with undiagnosed diabetes (Atlas, 2015; Saeedi et al., 2019). At present, insulin and oral hypoglycemic agents are used to treat diabetes. However, due to unwanted side effects of these currently used antidiabetic agents, WHO has recommended the investigation of new entities with lesser or no side‐effects for the treatment of diabetic patients (Gengiah et al., 2014).

Diabetes mellitus is a progressive metabolic disease marked by glucose, lipid, and lipoprotein defects, which results in hyperglycemia and induces numerous complications, such as hyperlipidemia and atherosclerosis (Ozder, 2014). Cholesterol is the cell membrane building block and a precursor to steroid hormones. It comprises of several different particulate substances, high‐density lipoproteins (HDLs), low‐density lipoproteins (LDLs), and very low‐density lipoprotein (VLDL). The LDL and VLDL cholesterol levels have been well known to be atherogenic, while HDL cholesterol protects the progression of atherosclerosis (Deng & Chow, 2010). Liver disorders that are now a worldwide health problem can be categorized as acute hepatitis, chronic hepatitis, hepatitis, and cirrhosis. Unfortunately, hepatitis therapy is controversial since generic or synthetic medications are not adequate to cure these diseases and often have significant side effects. Natural remedies have long proven effective for liver disorders (Asadi‐Samani et al., 2015). As the source of raw material for the extraction of secondary active metabolites and synthesis components, medicinal plants are widely used in modern drugs (Vijayakumar et al., 2020). The detoxification of the poisoned liver can be profoundly achieved through the use of natural ingredients, including herbal extracts. According to credible scientific data originating from medicinal plant research, plants such as *Glycyrrhiza glabra, Silybum marianum, Picrorhiza kurroa*, and Phyllanthus species have been extensively and regularly used to treat liver disorders (McBride et al., 2012; Tatiya et al., 2012). Mushrooms have a remarkable place in folk medicines not only for nutritional value but also for therapeutic uses (Yang et al., 2008). Mushrooms were used extensively for medicinal purposes and as flavorful foodstuffs. Mushrooms have emerged in recent years as a significant class of bioactive products, that is, (Jeong et al., 2004) immunological and hypoglycemic activities (Hao et al., 2020), and antitumor (Chen et al., 2020). Mushrooms contain several bioactive compounds that make sure appropriate metabolic functioning of metabolic organs such as endocrine glands, pancreas, and liver (Calvo et al., 2016). As a result, mushrooms have shown effectiveness in controlling both blood glucose levels and diabetic complications (Zhang et al., 2016). *Ganoderma applanatum* belongs to the family Ganodermaceae which is distributed throughout the world (Paterson, 2006). The different parts of the *G. applanatum* contain Applanatumin A, Applanatumol A, Applanatumol E, Applanatumol P, Applanatumol Q, Applanoxidic acid E, Cytosporone C, Nigragillin, Ganoapplanin, Myrocin C, Sphaeropsidin D, Comazaphilone D, Epoxy‐4,4,14‐trimethyl3,7,11,15,20‐pentaoxo‐pregnane, Applanoxidic acid (C‐G), Stemphone B, Condidymic acid, etc; as its chemical compositions (Elkhateeb et al., 2018; Li et al., 2016; Luo et al., 2015, 2016). Boundless traditional medicinal uses of *Ganoderma* species have attracted the pharmaceutical industry recently (Loyd et al., 2018). Several studies reported that *Ganoderma applanatum* has many medicinal properties such as antibacterial, antiviral, antitumor, antifibrotic, antiobesity, antioxidative, and immunomodulatory (Gao et al., 2019; Luo et al., 2017). The goal of the present research is therefore to study the effectiveness of *Ganoderma applanatum* as a possible natural oral hypoglycemic, hypolipidemic, and hepatoprotective agent, or functional food, in reducing hyperglycemia along with other vascular complications in diabetic rats by pharmacological and computational approaches.

## 2 MATERIALS AND METHODS

### 2.1 Drugs and chemicals

Analytical grade drugs and solvents were utilized in the study. Alloxan monohydrate and glibenclamide were purchased from Sigma Chemicals Co., USA and Square Pharmaceuticals Ltd., Bangladesh, respectively. 0.9% NaCl was purchased from Beximco Pharmaceuticals Ltd., Bangladesh. Other chemicals of analytical grade were supplied by the Department of Pharmacy, University of Chittagong.

### 2.2 Collection of mushroom

Fruiting bodies of wild *Ganoderma applanatum* were collected from the campus of the University of Chittagong, Chittagong. The samples were identified and authenticated by Dr. Shaikh Bokhtear Uddin, Department of Botany, University of Chittagong. The accession number for *Ganoderma applanatum is given* (2018/004/Fungi/CU/DP).

### 2.3 Extract preparation

The fruiting bodies were shade dried for 14 days. They were then oven dried for better grinding and finely powdered using an electric grinder. In an air‐tight container, the powder was stored. Weighed (200 g of the dried powder for methanolic extract and 200 g dried powder for aqueous extract) sample was soaked in 3 L of methanol and 3 L of distilled water respectively in clean, sterilized, and flat‐bottomed glass container for 14 days accompanying occasional stirring and agitation at room temperature. It was then filtered using filter papers (Whatman size no.1). The extracts were obtained by evaporation using a rotary evaporator. A gummy concentrated black color residue of methanolic extract and aqueous extract was found with a yield of 24.25 g and 21.98 g, respectively. These extracts were kept in tightly closed glass containers and stored in the refrigerator for further use.

### 2.4 Animals

Wistar albino rats of both sexes weighing 180–200 g were procured from BCSIR Laboratories, Chattogram. The animals were used in pharmacological and toxicological studies. They were housed in cages in well‐ventilated, room temperature conditions with a natural 12‐hr day‐night cycle at the Animal House of Department of Pharmacy, Faculty of Biological Science, University of Chittagong. They were fed a balanced rodent pellet diet. Tap water ad libitum was also provided throughout the experimental period. Water was changed and the cages were cleaned every day. Rats were allowed to acclimatize upon arrival for 2 weeks to laboratory conditions. Permission was granted by the Departmental Ethical Review Committee, Department of Pharmacy, University of Chittagong, before embarking on the animal studies.

### 2.5 Acute toxicity test

Overnight fasted healthy Wistar albino rats of either sex were divided into five groups (*n* = 5 in each group). 2 ml of typical saline solution in distilled water was administered orally in the animals of the control group. The rats of the remaining four groups orally received both extracts in increasing dose levels of 100, 400, 700, and 4,000 mg/kg body weight. According to guidelines No. 425 of the Organization for Economic Cooperation and Development, any change in rats' behavioral, neurological, and autonomic profile was observed consistently up to 72 hr. In‐depth care of animals is also taken for 7 days (OECD, O., 2001).

### 2.6 Induction of diabetes in rats

During 24 hr of fasting duration, animals could freely access the water. Overnight fasted albino rats were made diabetic by injecting with the freshly prepared solution of alloxan (120 mg/kg in normal saline 0.9% NaCl) intraperitoneally (Nabeel et al., 2010). Blood glucose level measured after 3 days. Animals with blood glucose levels equal to 200 mg/dl or above were considered diabetic and used in the experiment (Hassan et al., 2019).

### 2.7 Experimental design

A total of 42 animals (6 standard and 36 diabetic rats) were used in the experiment. The rats were divided into seven groups (I–VII) of six rats (*n* = 6). Group I (standard control) and Group II (alloxan‐induced diabetic control) received 1% tween 80 in normal saline (0.9% NaCl) i.p. for 9 days. Groups III and IV consisted of alloxan‐induced diabetic rats which received methanolic extract at a dose of 250 and 500 mg/kg i.p. respectively in the vehicle for 9 days. Group V and Group VI consisted of alloxan‐induced diabetic rats treated with aqueous extract at a dose of 250 and 500 mg/kg i.p. respectively in the vehicle for 9 days. Group VII consisted of alloxan‐induced diabetic rats which received glibenclamide 1 mg/kg i.p. for 9 days.

### 2.8 Determination of blood glucose levels

Fasting blood samples were collected from the tail vein of the rats on 1st, 4th, 7th, and 9th day. Blood glucose level was determined by using a one‐touch glucometer (Contour Net EZ). The food and water intake was monitored daily for each rat. The periodical body weight difference of the individual animals was also measured during nine experimental periods.

### 2.9 Preparation of serum for the test

After 9 days, the foods (except waters) were removed from the rats. The intraperitoneal injections of ketamine (50 mg/kg body weight, i.p.) have been administered to the rats and made them anesthetized. The blood samples were extracted and transferred to tubes directly from the rats via a cardiac puncture. At 3,000 rpm, blood was centrifuged for 10 min to extract and remove red blood cells (Jemai et al., 2009). Serum samples were extracted and collected by a micropipette and placed for analysis in the refrigerator.

### 2.10 Biochemical parameters

Automated biochemical analyzers were used for the determination of serum lipid profiles (total cholesterol, HDL, LDL, triglycerides), liver function parameters (total bilirubin, albumin, AST, ALT, ALP), and kidney function parameters (serum creatinine, serum urea, BUN).

### 2.11 Protocol of docking for molecular analysis

#### 2.11.1 Molecular analysis: compounds and target proteins

Three‐dimensional structure of the dual peroxisome proliferator‐activated receptor agonist protein (ppara/g) (PDB ID: 3G9E) (Bénardeau et al., 2009) for the antidiabetic analysis, farnesoid X receptor (FXR) agonist protein (PDB ID: 3OMM) (Richter et al., 2011) for the hypolipidemic assessment, hepatitis C virus NS3/4A protease inhibitors (PDB ID: 3SU4) (Romano et al., 2012) and human IgG Fc domain (PDB ID: 4QGT) (Chen et al., 2016; Ruan et al., 2019) for the hepatoprotective analysis have been retrieved from the protein data bank (Berman et al., 2000) in PDB format. Besides, the chemical compounds of *G. applanatum,* namely Applanoxidic acid E (PubChem CID: 10324125), Cytosporone C (PubChem CID: 10778975), Ganoapplanin (PubChem CID: 132581256), Nigragillin (PubChem CID: 15939563), Myrocin C (PubChem CID: 11067914) have been derived from the PubChem database (https://pubchem.ncbi.nlm.nih.gov/) (Wang et al., 2020) in SDF format.

#### 2.11.2 Molecular analysis: preparation of target protein and compounds

The ligands have been imported in 2DSDF format, and hereafter the ligands have been minimized and converted to pdbqt format across the PyRx tools to find the best hit in these targets. The protein structure was created with Discovery Studio and UCSF Chimera. Default settings in the PyRx from the MGL Tools (Herowati & Widodo, 2014) have been used for the virtual screening. Through the Discovery Studio 2020, all water and the heteroatom were eliminated from proteins. A combination of nonpolar hydrogen and the Gasteiger charge was used to assemble proteins. Besides, unnecessary solvents were deleted, and selenomethionine (MSE), to methionine (MET), Bromo UMP to UMP (U), methylselenyl‐dUMP (UMS), to UMP (U), methylselenyl‐dCMP (CSL) to CMP (C) have been marked to keep only highest occupancy. Again, the incomplete side chains were replaced by Dunbrack 2010 rotomer library. Furthermore, all proteins have been lowered to the least energy level by keeping the residues in AMBER ff14sB and Gasteiger mode in UCSF Chimera (Beglov et al., 2012).

#### 2.11.3 Molecular analysis: molecular docking

For the protein‐ligand binding operation of the selected protein‐ligand complexes, PyRx Autodock Vina was used (Emon, Alam, et al., 2020). A semiflexible docking system has been applied to perform the docking research. A semiflexible docking system has been applied to perform the docking research. The phytochemicals were translated into PDBQT formats with PyRx AutoDock tools. The rigidity of proteins and ligands was retained for this analysis. Ligand molecules had given the freedom for 10 degrees. AutoDock determines the molecules to format pdbqt, box style, grid box formation, etc. The grid box with an active position was built in the middle of the box. BIOVIA Discovery Studio Visualizer 2020 (Emon et al., 2021) was eventually accelerated to evaluate the docking sites for the possible linking approaches.

### 2.12 Statistical analysis

The data are presented as mean ±standard deviation (*SD*). Significance among different groups was determined using the one‐way analysis of variance (ANOVA) test, followed by Dunnet's *t* test (2‐sided). Values of *p* < .05, *p* < .01, *p* < .001 were considered as significant. All the data were analyzed using SPSS (Statistical Package for the Social Sciences) version 16.0 software. Charts were drawn using GraphPad Prism software version 6.01.

## 3 RESULTS

### 3.1 Acute toxicity test

The behavioral, neurological, and autonomic profiles of animals remained unchanged during this test duration. No death of rats was observed at the highest dose of both extracts.

### 3.2 Effect of MEGA and AEGA on blood glucose level

Intraperitoneal administration of a single dose of alloxan monohydrate (120 mg/kg) significantly (*p* < .001) increases the blood glucose level in diabetic control rats when compared with normal control rats. During the 9 days of treatment, there was a significant (*p* < .001) reduction in blood glucose level in Group III, Group IV, Group V, Group VI, and Group VII diabetic rats, which respectively received MEGA 250 mg/kg, MEGA 500 mg/kg, AEGA 250 mg/kg, AEGA 500 mg/kg, and glibenclamide 1 mg/kg body weight intraperitoneally as compared with alloxan‐induced untreated diabetic Group II rats (Table 1).

**TABLE 1:** Effect of MEGA and AEGA on blood glucose level in different groups of experimental rats

Groups	Blood glucose level (mg/dl)			
	Day 1	Day 4	Day 7	Day 9
I: Normal control	96.50 ± 3.73	99.83 ± 4.96	97.00 ± 3.16	100.17 ± 3.49
II: Diabetic control	285.83 ± 11.48^a^	290.50 ± 9.59^a^	303.50 ± 6.06^a^	305.83 ± 6.05^a^
III: Diabetic + MEGA (250 mg/kg)	135.83 ± 6.79^a^	127.83 ± 5.49^a^	119.17 ± 4.26^a^	108.33 ± 4.32^a^
IV: Diabetic + MEGA (500 mg/kg)	129.17 ± 2.99^a^	117.50 ± 10.03^a^	108.00 ± 3.03^a^	102.00 ± 3.69^a^
V: Diabetic + AEGA (250 mg/kg)	149.33 ± 4.18^a^	133.00 ± 5.69^a^	127.67 ± 5.96^a^	124.33 ± 4.89^a^
VI: Diabetic + AEGA (500 mg/kg)	142.17 ± 4.17^a^	127.67 ± 4.76^a^	124.33 ± 4.41^a^	110.33 ± 5.82^a^
VII: Diabetic + glibenclamide (1 mg/kg)	123.50 ± 3.73^a^	98.83 ± 7.63^a^	91.33 ± 4.27^a^	86.50 ± 6.22^a^

Note

All values are mean ± *SD* and statistically analyzed using one‐way analysis of variance (ANOVA) followed by Dunnett's multiple comparison test, *n* = 6. Group II compared with normal control. Groups III, IV, V, VI, and VII compared with diabetic control.

^a^ *p* < .001.

^b^ *p* < .01.

^c^ *p* < .05.

### 3.3 Effect of MEGA and AEGA on body weight

It was observed that the bodyweight of alloxan‐induced Group II diabetic animals gradually decreased when compared (*p* < .001) to the normal rats indicating the impaired glucose metabolism. After 9 days of treatment with the methanol extract (250 mg/kg; b.w) and aqueous extract (250 and 500 mg/kg; b.w) of *Ganoderma applanatum*, a significant gain in body weight was recognized in diabetic rats, and the results were compared with the diabetic control rats. After the 9th day, the body weight was elevated to 152.71 ± 1.52 which was further elevated to 164.70 ± 6.16 with the administration of AEGA 500 mg/kg; b.w. The observations have been summarized in Table 2.

**TABLE 2:** Effect of MEGA and AEGA on body weight in different groups of experimental rats

Groups	Body weight (gm)			
	Day 1	Day 4	Day 7	Day 9
I: Normal control	172.10 ± 3.23	175.70 ± 3.02	177.47 ± 3.20	178.20 ± 3.24
II: Diabetic control	157.90 ± 2.93^a^	156.83 ± 2.86^a^	154.11 ± 1.63^a^	152.71 ± 1.52^a^
III: Diabetic + MEGA (250 mg/kg)	158.60 ± 5.78	160.64 ± 5.41	161.06 ± 5.31^c^	161.48 ± 5.52^b^
IV: Diabetic + MEGA (500 mg/kg)	148.12 ± 3.32^b^	147.13 ± 3.18^b^	150.05 ± 2.61	152.14 ± 2.67
V: Diabetic + AEGA (250 mg/kg)	162.29 ± 4.55	161.14 ± 3.87	163.31 ± 4.54^b^	164.96 ± 3.83^a^
VI: Diabetic + AEGA (500 mg/kg)	159.47 ± 6.22	161.25 ± 6.09	163.40 ± 5.52^b^	164.70 ± 6.16^a^
VII: Diabetic + glibenclamide (1 mg/kg)	153.84 ± 3.60	157.39 ± 5.55	158.30 ± 5.05	159.19 ± 4.79

Note

All values are Mean ± *SD* and statistically analyzed using one‐way analysis of variance (ANOVA) followed by Dunnett's multiple comparison test, *n* = 6. Group II compared with normal control. Groups III, IV, V, VI, and VII compared with diabetic control.

^a^ *p* < .001.

^b^ *p* < .01.

^c^ *p* < .05.

### 3.4 Effect of MEGA and AEGA on serum lipid profile

In the control group, the level of the total cholesterol, HDL, LDL, and triglycerides was 71.67 ± 1.86, 28.00 ± 1.41, 88.00 ± 4.24, and 30.67 ± 2.58, respectively. The concentration of triglycerides and cholesterol was significantly (*p* < .001 for triglycerides and *p* < .05 for cholesterol) increased in Group II diabetic animals compared to Group I normal animals. The serum lipid profile of the control and the experimental animals has been depicted in Table 3.

**TABLE 3:** Effect of MEGA and AEGA on serum lipid profile in different groups of experimental rats

Groups	Parameters (mg/dl)			
	Total cholesterol	HDL	LDL	Triglycerides
I: Normal control	71.67 ± 1.86	28.00 ± 1.41	30.67 ± 2.58	88.00 ± 4.24
II: Diabetic control	66.83 ± 2.71^c^	30.5 ± 3.08	26.67 ± 3.01	107.50 ± 4.14^a^
III: Diabetic + MEGA (250 mg/kg)	54.00 ± 3.16^a^	25.83 ± 3.48	13.83 ± 2.04^a^	85.17 ± 1.72^a^
IV: Diabetic + MEGA (500 mg/kg)	48.83 ± 2.79^a^	26.83 ± 2.86	11.83 ± 2.32^a^	81.50 ± 2.74^a^
V: Diabetic + AEGA (250 mg/kg)	56.00 ± 2.28^a^	28.83 ± 3.13	17.67 ± 3.98^a^	89.17 ± 2.14^a^
VI: Diabetic + AEGA (500 mg/kg)	59.17 ± 2.93^a^	31.50 ± 1.87	15.33 ± 2.34^a^	82.83 ± 2.64^a^
VII: Diabetic + glibenclamide (1 mg/kg)	56.00 ± 3.46^a^	29.17 ± 3.97	17.83 ± 2.48^a^	91.50 ± 3.08^a^

Note

All values are Mean ± *SD* and statistically analyzed using one‐way analysis of variance (ANOVA) followed by Dunnett's multiple comparison test, *n* = 6. Group II compared with normal control. Groups III, IV, V, VI, and VII compared with diabetic control.

^a^ *p* < .001.

^b^ *p* < .01.

^c^ *p* < .05.

### 3.5 Effect of MEGA and AEGA on serum liver function parameters

The serum levels of liver enzymes such as AST, ALT, and ALP significantly (*p* < .001) increased in alloxan‐induced diabetic control rats as compared with normal control animals indicating hepatic damage. The markers of the AST, ALT, and ALP enzymes of the liver were decreased significantly (*p* < .001) with the administration of MEGA 250 mg/kg, MEGA 500 mg/kg, AEGA 250 mg/kg, and AEGA 500 mg/kg. The AST, ALT, and ALP were 52.33 ± 4.72, 126.33 ± 5.79, and 248.83 ± 5.46 in diabetic mice but with the administration of the MEGA 500 mg/kg, the level of AST, ALT and ALP was reduced to 25.00 ± 3.74, 98.00 ± 4.38, and 224.17 ± 3.87, respectively. The AST, ALT, and ALP also reduced to 30.00 ± 5.10, 98.83 ± 3.25, and 228.00 ± 5.22, respectively. The results of the extracts have been compared with the results of diabetes and glibenclamide 1 mg/kg induced mice. These findings proved the hepatoprotective effect of *Ganoderma applanatum* mushroom (Table 4).

**TABLE 4:** Effect of MEGA and AEGA on serum liver function parameters in different groups of experimental rats

Groups	TB (mg/dl)	Albumin (g/dl)	AST (U/L)	ALT (U/L)	ALP (U/L)
I: Normal control	1.00 ± 0.04	2.96 ± 0.03	38.50 ± 3.27	110.33 ± 6.09	216.67 ± 4.80
II: Diabetic control	1.38 ± 0.18^a^	2.87 ± 0.19	52.33 ± 4.72^a^	126.33 ± 5.79^a^	248.83 ± 5.46^a^
III: Diabetic + MEGA (250 mg/kg)	1.36 ± 0.11	2.80 ± 0.26	28.00 ± 4.05^a^	92.83 ± 3.54^a^	219.00 ± 4.73^a^
IV: Diabetic + MEGA (500 mg/kg)	1.35 ± 0.11	3.20 ± 0.28	25.00 ± 3.74^a^	98.00 ± 4.38^a^	224.17 ± 3.87^a^
V: Diabetic + AEGA (250 mg/kg)	1.36 ± 0.10	2.60 ± 0.42	34.00 ± 6.90^a^	94.17 ± 5.38^a^	222.00 ± 4.77^a^
VI: Diabetic + AEGA (500 mg/kg)	1.33 ± 0.13	3.20 ± 0.30	30.00 ± 5.10^a^	98.83 ± 3.25^a^	228.00 ± 5.22^a^
VII: Diabetic + glibenclamide (1 mg/kg)	1.36 ± 0.20	3.03 ± 0.34	22.00 ± 4.56^a^	87.00 ± 4.38^a^	162.50 ± 3.15^a^

Note

All values are Mean ± *SD* and statistically analyzed using one ‐way analysis of variance (ANOVA) followed by Dunnett's multiple comparison test, *n* = 6. Group II compared with normal control. Groups III, IV, V, VI, and VII compared with diabetic control.

^a^ *p* < .001.

^b^ *p* < .01.

^c^ *p* < .05.

### 3.6 Effect of MEGA and AEGA on serum kidney function parameters

Table 5 shows kidney function analysis including the study of creatinine, urea, and blood urea nitrogen (BUN). During the treatment, there was no significant effect on the kidney function parameters in diabetic control groups and the data were compared with the normal control group. Again, when the diabetic animals treated the test drugs, there was no significant effect on serum creatinine, serum urea, and BUN level observed and compared to the diabetic control group. With the administration of MEGA 500 mg/kg and AEGA 500 mg/kg, the serum urea were measured to 58.50 ± 5.75 (mg/dl) and 54.83 ± 4.54 (mg/dl). Besides, the results of BUN were yielded as 25.16 ± 1.18 (mg/dl) and 25.45 ± 1.10 (mg/dl) with the administration of MEGA and AEGA (500 mg/kg). So, the use of MEGA and AEGA did not damage the kidney.

**TABLE 5:** Effect of MEGA and AEGA on serum kidney function parameters in different groups of experimental rats

Groups	Parameters (mg/dl)		
	Serum creatinine	Serum urea	BUN
I: Normal control	0.91 ± 0.21	54.17 ± 4.49	25.39 ± 2.23
II: Diabetic control	0.88 ± 0.12	52.33 ± 5.47	24.30 ± 2.22
III: Diabetic + MEGA (250 mg/kg)	0.98 ± 0.15^b^	56.17 ± 6.11^b^	27.33 ± 2.99^b^
IV: Diabetic + MEGA (500 mg/kg)	0.93 ± 0.17^c^	58.50 ± 5.75^b^	25.16 ± 1.18
V: Diabetic + AEGA (250 mg/kg)	0.92 ± 0.11^c^	57.67 ± 7.42^b^	27.13 ± 1.23^b^
VI: Diabetic + AEGA (500 mg/kg)	0.88 ± 0.08	54.83 ± 4.54	25.45 ± 1.10
VII: Diabetic + glibenclamide (1 mg/kg)	0.86 ± 0.11	54.33 ± 8.29	25.20 ± 4.18

Note

All values are Mean ± SD and statistically analyzed using One Way Analysis of Variance (ANOVA) followed by Dunnett’s multiple comparison test, *n* = 6. Group II compared with normal control. Groups III, IV, V, VI and VII compared with diabetic control.

^a^ *p* < .001.

^b^ *p* < .01.

^c^ *p* < .05.

### 3.7 Molecular docking: antidiabetic, hypolipidemic, and hepatoprotective activities

**FIGURE 1:**
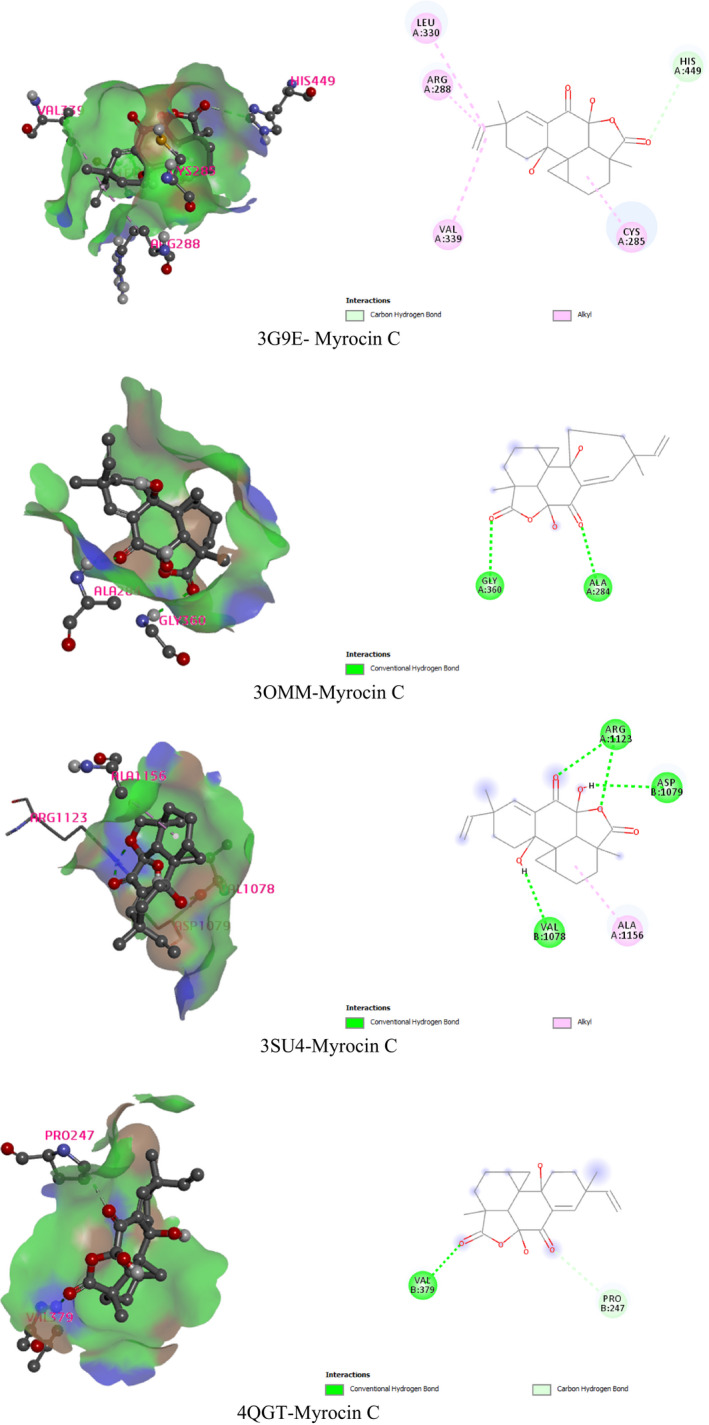
Best 2D and 3D pose of interacted molecules and proteins (3G9E‐Myrocin C, 3OMM‐Myrocin C, 3SU4‐Myrocin C, and 4QGT‐Myrocin C)

An effort to dock against analytical proteins in which all the compounds sought a better orientation and conjugation may interfere with the target protein. Entirely five (Applanoxidic acid E, Cytosporone C, Ganoapplanin, Nigragillin, and Myrocin C) bioactive constituents have been uncovered for the docking against dual ppara/g agonist protein (PDB ID: 3G9E) for the antidiabetic activity, farnesoid X receptor (FXR) agonist protein (PDB ID: 3OMM) for the hypolipidemic activity, hepatitis C virus NS3/4A protease inhibitors (PDB ID: 3SU4) and human IgG Fc domain (PDB ID: 4QGT) for the hepatoprotective activity. Initially, based on the scoring function, all compounds were screened. The glide score of Myrocin C was almost the highest for each selected protein. The Myrocin C interacted with val339, arg288, leu330, his449, cys285 residues of dual ppara/g agonist protein. This compound also reacted with gly360, ala284 residue of farnesoid X receptor (FXR) agonist protein. Besides, Myrocin C also interacts with the val1078, arg1123, asp1079, ala1156 and val379, pro247 residues of the hepatitis C virus NS3/4A protease inhibitors and human IgG Fc domain, respectively (Table 6 and Figure 1).

**TABLE 6:** Scores of binding affinity of the selected compounds and enzymes

Docking score					
Compounds	PubChem CID	Antidiabetic	Hypolipidemic	Hepatoprotective	
		3G9E	3OMM	3SU4	4QGT
Applanoxidic acid E	10324125	1.5	−8.5	−9.0	–
Cytosporone C	10778975	−7.8	−6.0	−5.6	−4.7
Myrocin C	132581256	−11.2	−10.6	−10.4	−8.6
Nigragillin	15939563	−7.2	−5.7	−6.0	−4.5
Ganoapplanin	11067914	−7.6	−7.5	−8.4	−6.7
Standard drugs (glyburide/lovastatin/betaine)	3488/53232/247	−9.3	−6.8	−3.6	−2.9

Note

PDB ID: 3G9E = Dual ppara/g agonist protein, PDB ID: 3OMM = farnesoid X receptor (FXR) agonist protein, PDB ID: 3SU4 = hepatitis C virus NS3/4A protease inhibitors and PDB ID: 4QGT: human IgG Fc domain.

## 4 DISCUSSION

The most potent source of new bioactive molecules for new therapies is medicinal plants (Alam et al., 2020). Therefore, natural plant‐based treatments are prevalent in developing countries and have essential priorities for their beneficial features for human health. Almost 80% of patients also use conventional drugs in developing countries (Kim, 2005). Various plant derivatives exert significant pharmacological actions, such as antioxidants, cytotoxicity, anxiolytics, thrombolytics, neuroprotective, antidepressants, hepatoprotective and neuroprotective actions (Okwu & Uchenna, 2009). Different countries have different remedy methods to resolve this, whereas developing countries use many techniques and symptomatic therapies. However, because of low costs, convenience, and accessibility, tribal groups favor conventional drugs as their primary health care system (Chowdhury et al., 2020). According to (Akindele et al., 2015), patients should use integrative therapy if their disorder does not respond or minimize synthetic medicines. In past studies, many mushrooms such as (*Pleurotus ostreatus, Lignosus rhinocerotis, Pleurotus salmoneostramineus, Auricularia auricula‐judae, Ganoderma lucidum*, *Phyllantus amarus, Tremella fuciformis, Auricularia auricular, Flammulina velutipes, Lentinula edodes, Volvariella volvacea, Tricholoma lobayense,* and *Grifola frondosa*) were discovered in various ways to tackle diabetes (Ravi et al., 2013; De Silva et al., 2012), hyperlipidemic (Liang et al., 2018), and hepatic (Liu et al., 2017; Soares et al., 2013) degeneration. Our research is the first systematic pharmacological and computational assessment, including antidiabetic, hypolipidemic, and hepatoprotective effects of methanol and aqueous extracts of the *Ganoderma applanatum* (MEGA and AEGA). The experimental rats showed an average basal blood glucose level before the administration of alloxan. Alloxan is a diabetogenic agent that destroys the islets of Langerhans, resulting from a massive reduction in insulin release, causing hyperglycemia (2016). In the present investigation, it was observed that these extracts were able to reduce the blood glucose level from the day‐1 of its administration, indicating the strong hypoglycemic tendency of the phytoconstituents present in the extracts. *Ganoderma applanatum* might aid in the recovery of β cells to secrete insulin; therefore, the blood glucose level was decreased after treatment. It was an indication that the drug treatment ameliorated the impaired carbohydrate metabolism. Induction of diabetes by alloxan leads to loss of body weight due to increased muscle wasting and loss of tissue proteins (Obia et al., 2016). But, the body weights of the rats have been increased after the administration of MEGA and AEGA. The phytochemicals in the mushroom extract might enhance glucose utilization and improve the diabetes‐associated complications, thereby restoring the body homeostasis and increasing body weight (Zhao et al., 2019).

Cholesterol levels hugely influence the cardiovascular disease process. Hyperlipidemia is known as an increase in lipids level, including serum cholesterol and serum triglycerides which is highly responsible for the development of atherosclerosis (McGinty & Siddiqui, 2020). The consumption of mushrooms markedly decreased the lipid level (de Miranda et al., 2017; Jeong et al., 2010). Results obtained in this study represent that both extracts of *Ganoderma applanatum* mushroom (MEGA 250 mg/kg, MEGA 500 mg/kg, AEGA 250 mg/kg and AEGA 500 mg/kg body weight) along with glibenclamide (1 mg/kg body weight) caused significant (*p* < .001) reduction in serum cholesterol, LDL cholesterol, and triglycerides. The suppression of LDL oxidation by *Ganoderma applanatum* may reduce the serum LDL significantly. Low triglyceride levels can contribute to decreasing the availability of fatty acids for esterification (Packard et al., 2020), increased catabolism of LDL, activation of tissue lipases (McCarty, 2001), and decreased production of precursors of triglycerides (Packard et al., 2020). This observed restoration of the alloxan‐evoked changes in the serum lipid profile shows the protective nature and hypolipidemic effect of *Ganoderma applanatum*. These results suggest that *Ganoderma applanatum* can be used for the management of hyperlipidemia and atherosclerosis. The bioactive components for hypolipidemic activity in *Ganoderma applanatum* are yet to be identified. In this evaluation, MEGA and AEGA have shown that they can bind bile acids with cholesterol metabolites and lower the solubility of cholesterol. After administering MEGA and AEGA to the rats, the fecal excretion of cholesterol and bile acid has increased significantly. The suggestion is that reductions in intestinal cholesterol and bile acid absorption after the feeding of MEGA and AEGA may represent a mechanism for MEGA and AEGA's hypolipidemic mechanisms (Nagaoka et al., 2005). Injury to the structural integrity of the liver is seen by an elevated serum transaminase as the cytoplasms are found and discharged into circulation after cellular damage (Pari & Kumar, 2002). Enhancement of bilirubin indicates abnormal liver function, resulting from the higher synthetic function of the liver (Nasrin et al., 2011). In this study, Table 4 shows a significant (*p* < .001) rise in the bilirubin level of diabetic animals compared with normal control animals. But *Ganoderma applanatum* mushroom did not affect albumin production and bilirubin level in diabetic rats. Diabetic complications such as increased gluconeogenesis and ketogenesis may be due to the elevated enzymes (Kumar et al., 2011). The liver mainly detoxifies xenobiotics. Elevated liver enzymes act as a mediator for liver damage (Hasan et al., 2018). Table 5 shows kidney function analysis including the study of creatinine, urea, and blood urea nitrogen (BUN). Elevation of renal function markers such as plasma levels of urea and creatinine is considered as renal dysfunction or kidney disease (Gowda et al., 2010). There was no significant effect on the kidney function parameters in diabetic control groups during the treatment than the standard control group. Again, when the diabetic animals treated with the test drugs, there was no significant effect on serum creatinine, serum urea, and BUN level observed in comparison with the diabetic control group. So, the use of *Ganoderma applanatum* did not damage the kidney. Molecular docking analysis is a systemic way to predict the ligand‐protein relationship that is most suitable for gaining knowledge about the biological activity of the bioactive components. Yet to know more details on the basis of possible modes of action and binding interaction between the ligands and different binding sites of protein molecules (Emon, Jahan, et al., 2020). To forecast the conceivable biological (antidiabetic, hypolipidemic and hepatoprotective) mode of actions of MEGA and AEGA, Applanoxidic acid E, Cytosporone C, Ganoapplanin, Nigragillin, Myrocin C, and Standard drug (Glyburide) have been chosen for the docking studies, whereas the dual ppara/g agonist protein (PDB ID: 3G9E) (Bénardeau et al., 2009), farnesoid X receptor (FXR) agonist protein (PDB ID: 3OMM) (Richter et al., 2011), hepatitis C virus NS3/4A protease inhibitors (PDB ID: 3SU4) (Romano et al., 2012), and Human IgG Fc Domain (PDB ID: 4QGT) (Chen et al., 2016) have been used. From the results of the molecular docking, it can be inferred that the bioactive compounds of *G. applanatum* have auspicious binging affinity toward dual ppara/g agonist protein, farnesoid X receptor (FXR) agonist protein, hepatitis C virus NS3/4A protease inhibitors, and human IgG Fc domain receptor. The pharmacological and computational profiles of the *G*. *applanatum* are found good with very mild toxicity, which is essential to be a possible drug.

## 5 CONCLUSION

The pharmacological results of the present study exhibited that *Ganoderma applanatum* mushroom exerts antidiabetic, hypolipidemic, and hepatoprotective effects that are also validated and showed by computational investigations. This could be due to the presence of different types of active constituents in this mushroom. So, *Ganoderma applanatum* mushroom may serve as an antidiabetic, hypolipidemic, and hepatoprotective agent to diabetic, hyperlipidemia, and hepatic patients with high glucose level and other diabetic complications.

## CONFLICT OF INTEREST

The authors declare no known conflict of interest to any organization or individuals.

## Data Availability

All the data have been incorporated into the manuscript.
